# Trusses and Stockings

**Published:** 1887-04-16

**Authors:** 


### XVI.—TRUSSES AND STOCKINGS.

*Trusses.*—Are used when a part of the bowel protrudes at a weak part of the abdominal wall. It is necessary to be careful in many details as to their application. Thus the rupture, as it is called, always tends to come down more when the patient is standing or sitting than when lying; and as the truss is used to keep it in place, and must only be put on when the bowel is quite returned, it must be taken off last thing at night, when the patient is lying down, and replaced in the morning before rising, after seeing that the bowel is returned. The skin also must be carefully sponged and cleansed every night, and a little starch powder applied. Care must be taken that the truss fits evenly, without pressing particularly on any one point, so as to cause a sore.

*Elastic Stockings.*—Much the same rules apply to these as to trusses. Thus they must be removed at night when in bed, and put on again in the morning before rising, or hanging down the legs at all. It is also advisable to carefully stroke the veins upwards from the foot to the thigh before applying the stockings.

(*To be continued.*)

### TWO PHYSICIANS BETTER THAN ONE

A Challenge.

*A Layman:*

One prompt physician like a sculler plies,
And all his art and all his skill applies:
But two physicians, like a pair of oars,
Convey you soonest to the Stygian shores.

### ONE PHYSICIAN BETTER THAN NONE.

A Rejoinder.

*The Doctor:*

When men are sick, the doctor "quick"
They send for to attend 'em;
By wit and skill, or cunning pill,
He promptly doth amend 'em.

Once out of doors, to settle scores
With Doctor, *some abuse him;*
But wiser men look forward when
*Again they'll want to use him.*

These gratefully send lib'ral fee,
"With thanks for kind attention."
This, truth to say's the better way:
And needs no further mention.

